# Physiologic and Metabolic Changes in *Crepidiastrum denticulatum* According to Different Energy Levels of UV-B Radiation

**DOI:** 10.3390/ijms21197134

**Published:** 2020-09-27

**Authors:** Song-Yi Park, Mee-Youn Lee, Choong-Hwan Lee, Myung-Min Oh

**Affiliations:** 1 Division of Animal, Horticultural, and Food Sciences, Chungbuk National University, Cheongju 28644, Korea; 1songyi1@gmail.com; 2 Brain Korea 21 Center for Bio-Health Industry, Chungbuk National University, Cheongju 28644, Korea; 3 Department of Bioscience and Biotechnology, Konkuk University, Seoul 05029, Korea; kkamlice@hanmail.net (M.-Y.L.); chlee123@konkuk.ac.kr (C.-H.L.)

**Keywords:** medicinal plants, physical elicitors, maximum quantum yield of photosystem II, antioxidant phenolic compounds, metabolite profiles

## Abstract

Ultraviolet B (UV-B) light, as a physical elicitor, can promote the secondary metabolites biosynthesis in plants. We investigated effects of different energy levels of UV-B radiation on growth and bioactive compounds of *Crepidiastrum denticulatum*. Three-week-old seedlings were grown in a plant factory for 5 weeks. Plants were subjected to different levels of UV-B (0, 0.1, 0.25, 0.5, 1.0, and 1.25 W m^−2^), 6 h a day for 6 days. All UV-B treatments had no negative effect on the shoot dry weight; however, relatively high energy treatments (1.0 and 1.25 W m^−2^) inhibited the shoot fresh weight. UV-B light of 0.1, 0.25, and 0.5 W m^−2^ did not affect total chlorophyll and H_2_O_2_ contents; however, they increased total carotenoid content. On 4 days, 0.25 W m^−2^ treatment increased antioxidant capacity, total hydroxycinnamic acids (HCAs) content, and several sesquiterpenes. Treatments with 1.0 and 1.25 W m^−2^ increased total carotenoid, total HCAs, and H_2_O_2_ contents, and destroyed chlorophyll pigments, reducing maximum quantum yield of photosystem II and causing visible damage to leaves. Partial least squares discrimination analysis (PLS-DA) showed that secondary metabolites were distinguishably changed according to energy levels of UV-B. The potential of 0.25 W m^−2^ UV-B for the efficient production of bioactive compounds without growth inhibition in *C. denticulatum* was identified.

## 1. Introduction

Ultraviolet B (UV-B) light, ranging from 280 to 320 nm, belongs to non-visible light spectra, and accounts for approximately 0.5% of the total solar light reached at Earth surface. It has the potential to alter morphological, physiological, biochemical, and genetic properties of plants, and can induce oxidative stress in plants due to its strong energy [1,2,3,4]. As the ozone layer is depleted, increasing amounts of UV-B radiation are reaching the Earth’s surface, and studies on UV-B perception and signaling in plants have been actively conducted for the last 20 years [1,5,6,7]. These studies have demonstrated that high energy UV-B radiation generates reactive oxygen species (ROS) in chloroplast, mitochondria, nucleus, and apoplast, damaging DNA, protein, cell membrane, and chloroplast, subsequently inhibiting plant growth. ROS generated by UV-B radiation promotes the biosynthesis of ROS scavenging enzymes (catalase, peroxidase, ascorbate peroxidase, etc.) and antioxidant compounds (ascorbic acid, glutathione, etc.), by mediating rapid systemic signaling involved in plant defense [8]. In addition, UV Resistance Locus8 (UVR8) initiates the stress signaling response by receiving UV-B light [2,7,9,10]. Among UV-B stress responses, an important change in terms of secondary metabolites is the accumulation of phenolic compounds, such as flavonoids, anthocyanins, and hydroxycinnamic acids (HCAs), that function as sunscreens in vacuoles of epidermal cells to protect important subcellular components from UV-B light [11,12,13]. However, plant responses to UV-B light depend on its energy level, duration, and peak wavelength and on the plant species, varieties, and growth stages [14,15,16,17]. Therefore, UV-B radiation with appropriate conditions can induce mass production of the aforementioned phenolic compounds in plants.

Of more than 500 phytochemicals biosynthesized by plants, bioactive compounds are defined as biomolecules that are beneficial to human health due to their ability to regulate various metabolic processes and pathways in the human body, including disease prevention, biorhythm control, and aging control [18]. Extracts from medicinal plants are widely used as raw materials for pharmaceuticals, flavors, fragrances, cosmetics, biopesticides, and food additives, as they are rich in bioactive compounds [18,19]. The annual growth rate of the global medicinal plant market has been increasing, and the market size is expected to reach approximately $129 million by 2023 [20,21]. Therefore, the medicinal plant-based industry is emerging as a promising field, with the market demonstrating a tremendous potential for economic growth.

*Crepidiastrum denticulatum* (Houtt.) Pak & Kawano, a native Korean medicinal plant, contains a plentiful amount of bioactive compounds, including various types of HCAs, that exert a hepatoprotective effect, and hence, *C. denticulatum* extract is used as a raw material for functional foods to improve liver function [22,23,24,25]. Most medicinal plants used in pharmaceuticals have been cultivated and harvested in fields so far. The quality of bioactive compounds obtained from medicinal plants grown outdoors is inconsistent due to various factors such as factions in absence of genetic fixation and changes in environmental conditions. In addition, natural habitats of medicinal plants are being destroyed because of indiscriminate land use and abnormal climates caused by global warming [26,27]. Thus, continuous and stable production of medicinal plants with consistent levels of target bioactive compounds is required for their application in the pharmaceutical industry.

The production of medicinal plants in a controlled environment, such as plant factories with artificial lighting (PFALs), not only overcomes the aforementioned problems, but also enables the enhancement of plant bioactive compounds using physical elicitors, such as UV-B light. In this study, we hypothesized that UV-B radiation at a certain energy level would increase the bioactive compound content in *C. denticulatum*. To confirm this hypothesis, *C. denticulatum* cultivated in PFAL was irradiated with UV-B light at different energy levels for 6 days, 1 week before harvesting, and changes in growth and the bioactive compound content were investigated.

## 2. Results

### 2.1. Shoot Biomass

**Figure 1:**
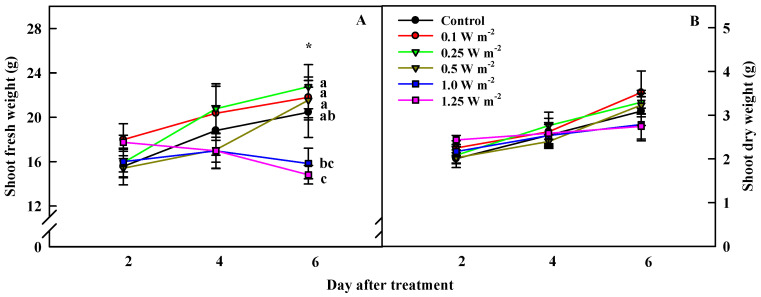
Shoot fresh (**A**) and dry (**B**) weights of *C. denticulatum* subjected to five energy levels of UV-B radiation for 6 days. The UV-B treatments started after 5 weeks of transplanting. Different letters next to bars indicate a significant difference by Duncan’s multiple range test at *p* < 0.05 (*n* = 12).

**Figure 2:**
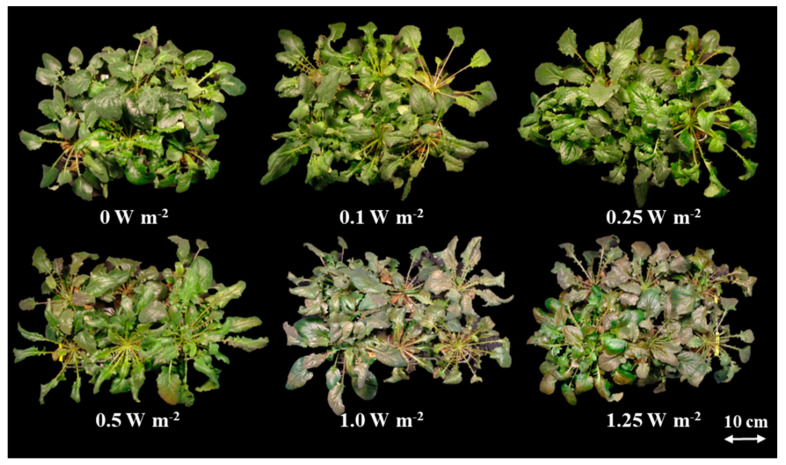
*C. denticulatum* subjected to five energy levels of UV-B radiation at 6 days after treatment. The UV-B treatments started after 5 weeks of transplanting.

Changes in shoot fresh and dry weights of *C. denticulatum* varied depending on the energy level of the UV-B radiation applied (Figure 1). There were no significant differences in the shoot fresh weight of the control (0 W m^−2^) and UV-B-irradiated plants, on days 2 and 4 of the treatment. However, the shoot fresh weight in 1.25 W m^−2^ treatment, which received the highest energy, was the lowest at 6 days of treatment, and that in 1.0 W m^−2^ treatment, was significantly lower than in the other three UV-B treatments. In contrast, no negative effects of UV-B radiation on the shoot dry weight were observed (Figure 1B). However, plants under 1.0 and 1.25 W m^−2^ treatments showed considerable browning and curling of leaves at 6 days of UV-B treatment (Figure 2).

### 2.2. Maximum Quantum Yield of Photosystem II and H_2_O_2_ Content

**Figure 3:**
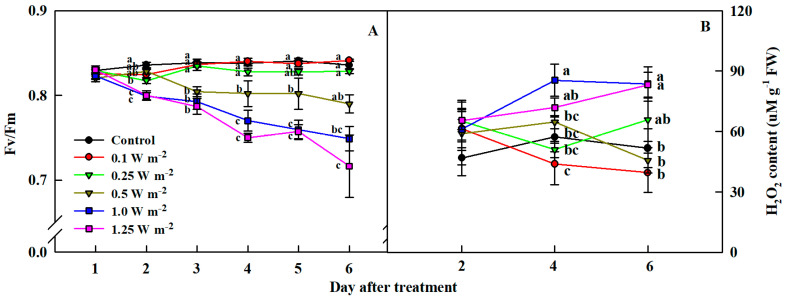
Maximum quantum yield of photosystem II (Fv/Fm) (**A**) and H_2_O_2_ content (**B**) of *C. denticulatum* subjected to five energy levels of UV-B radiation for 6 days. The UV-B treatments started after 5 weeks of transplanting. Different letters next to bars indicate a significant difference by Duncan’s multiple range test at *p* < 0.01 (Fv/Fm, *n* = 5; H_2_O_2_ content, *n* = 6).

As UV-B energy levels increased, the maximum quantum yield of photosystem II (Fv/Fm) significantly decreased (Figure 3A). Fv/Fm value in 0.25 W m^−2^ decreased to 0.81 at 2 days of the treatment, which was significantly different from that of the control, however, it recovered to 0.83 on day 3 of the treatment. From day 3 of UV-B treatment, Fv/Fm value in the 0.5 W m^−2^ treatment decreased to less than 0.8, and it was significantly different from that of the control on days 3, 4, and 5 of the treatment. Fv/Fm values in the 1.0 and 1.25 W m^−2^ treatments, with relatively high energy, were significantly different from the control and other UV-B treatments from 2 days, falling below 0.8 on day 3, and subsequently dropping to 7.4 and 7.1, respectively, until day 6 of UV-B treatment.

The content of H_2_O_2_, a representative ROS, significantly changed from day 4 of UV-B treatment (Figure 3B). H_2_O_2_ content in the 1.0 W m^−2^ treatment was significantly higher than that in the control (0 W m^−2^) and other UV-B treatments on day 4 of UV-B treatment. H_2_O_2_ contents in the 1.0 and 1.25 W m^−2^ were 1.6 times higher than that in the control on 6 days of UV-B treatment. However, H_2_O_2_ contents in the control and the other three UV-B treatments were not significantly different.

### 2.3. Total Chlorophyll and Carotenoid Contents

**Figure 4:**
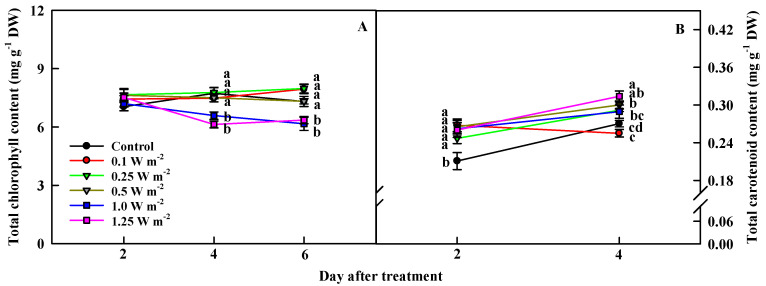
Total chlorophyll (**A**) and total carotenoid (**B**) contents of *C. denticulatum* subjected to five energy levels of UV-B radiation for 6 days. The UV-B treatments started after 5 weeks of transplanting. Different letters next to bars indicate a significant difference by Duncan’s multiple range test at *p* < 0.01 (*n* = 12).

Significantly lower total chlorophyll contents were recorded in the 1.0 and 1.25 W m^−2^ treatments, compared to the other UV-B treatments and the control (0 W m^−2^), on days 4 and 6 of UV-B treatment (Figure 4A). In contrast, total carotenoid content increased in response to UV-B radiation, from 2 days of UV-B treatment (Figure 4B). Total carotenoid content was significantly higher in all UV-B treatments than in the control. On 4 days of treatment, total carotenoid content in the 0.1 W m^−2^, i.e., the lowest energy treatment, had the lowest value, and that of 1.25 W m^−2^, i.e., the highest energy treatment, exhibited the highest value of 0.31 mg g^−1^, which was 20% higher than that of the control.

### 2.4. Total Phenolic Content and Antioxidant Capacity

**Figure 5:**
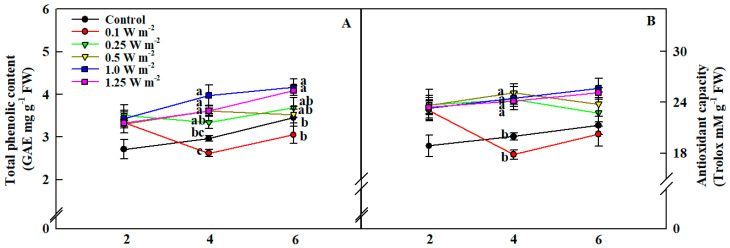
Total phenolic content (**A**) and antioxidant capacity (**B**) per unit fresh weight of *C. denticulatum* subjected to five different energy of UV-B radiation for 6 days. The UV-B treatments started after 5 weeks of transplanting. Different letters above bars indicate a significant difference by Duncan’s multiple range test at *p* < 0.05 (*n* = 6).

Total phenolic content and antioxidant capacity of *C. denticulatum* shoots were affected by different energy levels and duration of UV-B treatment (Figure 5). On the second day of treatment, total phenolic content and antioxidant capacity per unit fresh weight in UV-B treatments showed higher tendency compared to the control (0 W m^−2^), although they were not significantly different. At 4 days of treatment, there was a pronounced difference between the control and five UV-B treatments. Total phenolic contents in the 0.5, 1.0, and 1.25 W m^−2^ treatments were significantly higher, by at least 1.22 times, than that in the control, and all UV-B treatments, except for 0.10 W m^−2^, significantly increased antioxidant capacity compared to the control. Total phenolic contents in 1.0 and 1.25 W m^−2^ treatments were higher than that in the control at 6 days of UV-B treatment while there was no significant difference in antioxidant capacity.

### 2.5. Hydroxycinnamic Acids (HCAs)

Five different HCAs were identified in *C. denticulatum* shoots (Table 1). Only caffeic acid content of plants subjected to 0.25, 1.0, and 1.25 W m^−2^ UV-B treatments was significantly higher than that of the control (0 W m^−2^) at 2 days of treatment. Total HCA content was significantly higher in 1.0 W m^−2^ than in all other UV-B treatments, except for 1.25 W m^−2^ treatment. In particular, total HCAs content in 1.0 W m^−2^ treatment was 1.4 times higher than that of the control. However, on day 4 of UV-B treatment, caftaric acid, chicoric acid, and total HCA contents were the highest in 0.25 W m^−2^ treatment. Caffeic acid content showed the greatest increase in 0.25 W m^−2^ treatment, and was significantly higher than in the control. Caftaric acid and total HCA contents varied significantly between different energy levels and treatment duration of UV-B, and also exhibited a significant interaction coefficient between two factors was observed at least *p* < 0.05. Chicoric acid content was more affected by UV-B treatment duration than energy levels of UV-B, and an interaction effect between these two factors was detected. Duration and energy levels of UV-B had a significant impact on caffeic acid content, respectively, but no interaction effect was observed between the two factors. Conversely, chlorogenic acid and 3,5-di-O-caffeoylquinic acid (3,5-DCQA) contents showed no significant difference between UV-B treatments.

### 2.6. Metabolite Profiling

**Figure 6:**
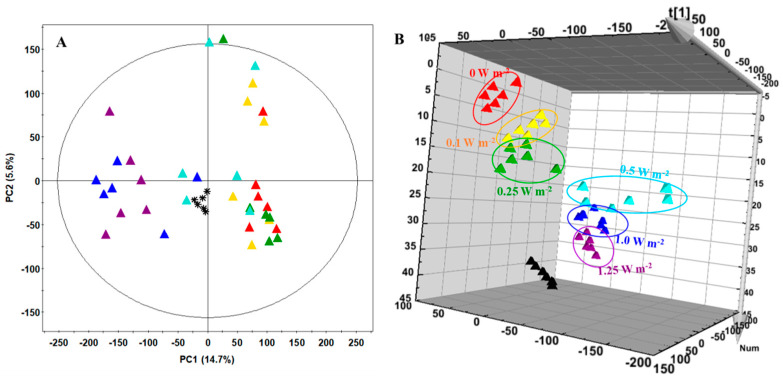
Principal component analysis (PCA) (**A**) and partial least squares discrimination analysis (PLS-DA) (**B**) score plots analyzed by ultra high performance liquid chromatography-linear ion trap-Orbitrap mass spectrometer (UPLC-LTQ-Orbitrap-MS) of *C. denticulatum* subjected to different energy levels of UV-B radiation for 4 days. ▲, 0 W m^−2^; ▲, 0.1 W m^−2^; ▲, 0.25 W m^−2^; ▲, 0.5 W m^−2^; ▲, 1.0 W m^−2^; ▲, 1.25 W m^−2^; * and ▲, quality control (QC).

**Figure 7:**
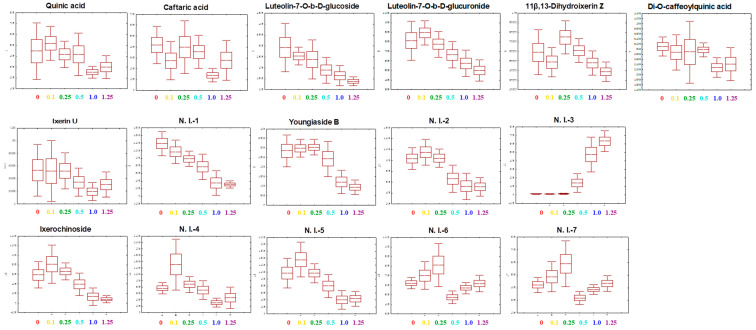
Box-and-whisker plots of metabolites in *C. denticulatum* subjected to different energy levels of UV-B radiation at 4 days after treatment using ultra high performance liquid chromatography-linear ion trap-Orbitrap mass spectrometer (UPLC-LTQ-Orbitrap-MS).

Metabolite profiles for the *C. denticulatum* by different energy levels of UV-B light were performed by ultra high performance liquid chromatography-linear ion trap-Orbitrap mass spectrometer (UHPLC-LTQ-Orbitrap-MS) on 4 days of UV-B treatment (Table S1). Principal component analysis (PCA) and partial least squares discrimination analysis (PLS-DA) analyses were used to distinguish the differences between groups and to interpret the intrinsic similarities of each group from their chromatographic profiles (Figure 6). The PLS-DA models obtained the metabolites gradually changed according to the energy level. The patterns of the metabolite profiles for the different energy levels of *C. denticulatum* clustered the six experimental groups. As shown in Figure 6B, metabolite profiles of 4 groups (0, 0.1, 0.25, and 0.5 W m^−2^) were separated by PLS1 (14.6%) and the 2 groups (1.0 and 1.25 W m^−2^) were separated by PLS2 (4.1%). The observed satisfaction values of X and Y variables in the PLS-DA model were 0.188 (R^2^X) and 0.288 (R^2^Y), respectively, with a prediction accuracy of 0.128 (Q^2^). The significantly discriminant metabolites corresponding to energy levels of UV-B radiation were determined using the variable importance in projection (VIP) value (VIP > 0.7) (Figure 7). Total of 16 were tentatively identified using measured mass, retention time, elemental composition, error (ppm), and mass spectrometry (MS^n^) fragments by references (Table S1). Among them, phenolic compounds were identified, including phenolic acids, flavonoids, and terpenoids. Quinic acid, luteolin-7-O-b-D-glucoside, luteolin-7-O-b-D-glucuronide, di-O-caffeoylqunic acid, youngiaside B, ixerin U, and ixerochinoside were slightly increased in 0.1 and 0.25 W m^−2^, and then were generally decreased with increasing energy levels (Figure 7). Some metabolites such as 11β,13-dihydroixerin Z, youngiaside B, and non-identified compounds (N.I.-6, 7) in 0.25 W m^−2^ were the highest values among all UV-B treatments and the control. Whereas contrary results were observed depending on the energy level based on 0.5 W m^−2^ in metabolites profiles.

## 3. Discussion

It is known that exposure to high UV-B energy acts as a distress and negatively affects plant growth via ROS-mediated signaling pathway. Conversely, low UV-B energy acts as a eustress, has less harmful effects on plant growth, and renders plants tolerance against stress by increasing leaf thickness and activating defense mechanisms via specific UVR8 response pathway [6,9,28]. Such conflicting results have been reported in many previous studies focusing on UV-B [6,28,29,30,31,32,33]. Exposure to a UV-B dose of 1.2, 4.3, 5.0, and 8.5 kJ d^−1^ was not found to affect growth in sunflower (*Helianthus annuus*), rosemary (*Rosmarinus officinalis*), soy bean (*Glycine max*), and basil (*Ocimum basilicum*), respectively [6,28,30,33]. However, biomass accumulation was inhibited in sugar beet (*Beta vulgaris*), soy bean and Arabidopsis, and maca (*Lepidium meyenii*), under 9.1, 10.0, and 18.2 kJ d^−1^ of UV-B, respectively [28,29,31,32]. UV-B energy levels of 0.1 W m^−2^ (2.4 kJ d^−1^), 0.25 W m^−2^ (5.2 kJ d^−1^), and 0.5 W m^−2^ (10.8 kJ d^−1^) used in this study did not damage the shoot biomass of *C. denticulatum*, however, 1.0 W m^−2^ (21.8 kJ d^−1^) and 1.25 W m^−2^ (27.2 kJ d^−1^) treatments inhibited the increase in shoot fresh weight and induced browning of leaves, indicating plant injury (Figure 1 and Figure 2 and Table 2). However, the shoot dry weight was not inhibited by UV-B radiation, suggesting that water loss in leaves was the main reason for the inhibition of shoot fresh weight accumulation. UV-B radiation destroys cell membranes, causing ions leakage, and subsequently inducing water evaporation, resulting in reduced fractional volume of mesophyll and guard cells [34].

When plants are adapted to darkness, the primary acceptor of photosysem II (PSII), i.e., quinone (Q_A_), is completely reduced, and the maximum quantum yield of PSII can be obtained by measuring Fo and Fm immediately before and after the saturation light, respectively [35]. These indexes can be used to measure the quantum yield efficiency of PSII when plants are irradiated using light sources. Under stress, Q_A_ cannot accept more electrons, which results in closing of the reaction center and decreasing in Fv/Fm value. O_2_^−^ and H_2_O_2_, which are representative ROS produced by UV-B radiation in chloroplasts, interfere with the light reaction process by oxidizing tryptophan, a component of D1 protein in PSII. Thus, UV-B light reduces the quantum efficiency of PSII and chlorophyll fluorescence value. In this study, Fv/Fm values of *C. denticulatum* leaves decreased continuously with increasing energy level and duration of UV-B exposure (Figure 3A). In addition, H_2_O_2_ content in the 1.0 and 1.25 W m^−2^ treatments, relatively strong energy treatments, tended to increase as the duration of UV-B exposure increased (Figure 3B). Since H_2_O_2_ content and Fv/Fm value exhibited a significant negative correlation (Table 3), it is likely that H_2_O_2_ produced by UV-B light exposure contributed to the reduction of the maximum quantum yield of PSII.

Photosynthetic pigments, such as chlorophylls and carotenoids, are sensitive to UV-B light, and are thus useful indicators of UV-B tolerance in plants [33,36,37]. When UV-B light is directly absorbed, chlorophylls can be decomposed into Mg^2+^ ion and pheophytin or it may be structurally broken down by ROS generated, which will subsequently lead to a reduced quantum efficiency of PSII [35]. This further causes the plant to dissipate excess light energy via non-photochemical quenching (NPQ), a photoprotective strategy to protect photosynthetic apparatuses. For example, xanthophyll cycle emits light energy as heat by converting it into various carotenoid pigments, such as zeaxanthin, antheraxanthin, and violaxanthin, using light energy [17,37]. In this study, 1.0 and 1.25 W m^−2^ treatments resulted in decreased the chlorophyll content and Fv/Fm values and increased total carotenoid content, on days 4 and 6 of UV-B treatment. Consequently, Fv/Fm value had a positive correlation with the chlorophyll content and a negative correlation with total carotenoid content (Table 3). This implies that high energy UV-B treatments destroyed chlorophylls, reducing the maximum quantum yield of PSII, while increasing total carotenoid content as a light protection strategy.

UV-B can accelerate the process of secondary metabolite biosynthesis in two ways. First, UVR8 dimer is converted to monomers by low-energy UV-B light, and the monomer then combines with CONSTITUTIVELY PHOTOMORPHOGENIC1 (COP1) to form a complex that stimulates ELONGATED HYPOCOTYL5 (HY5) transcription factor to activate stress defense responses. This leads to promote the biosynthesis of UV-B absorbing compounds, such as flavonoids, anthocyanins, and HCAs, which act as a sunscreen [9,10,35,36,37,38]. In addition, HY5 regulates a number of genes involving in the terpene biosynthesis pathway [39], and [40,41] reported that the relatively low UV-B radiation with 4.75 kJ d^−1^ induced the sesquiterpenes biosynthesis related to cell membrane stability, protecting leaves from UV-B-induced rapid heating. These compounds synthesized via UVR8 pathway are involved in protective responses against environment-induced oxidative stress because of their high antioxidant capacity [35,36,37,38,42]. Secondly, ROS produced in chloroplasts, mitochondrias, and apoplasts due to high level of UV-B light generate ROS waves, which mediate rapid systemic signaling, activating ROS-scavenging pathway to stimulate the biosynthesis of antioxidant secondary metabolites [8,9,10]. If stress persists or stress level is high, the accumulation of ROS generated from these pathways will be greater than that of antioxidants level, which subsequently damages plants [7,8,9]. In this study, all things considered, it suggests that 0.1 and 0.25 W m^−2^ UV-B might activate UVR8 pathway, as an eustress, to induce an increase in antioxidant capacity, HCAs content, and sesquiterpenes content without growth inhibition, whereas 1.0 and 1.25 m^−2^ UV-B might activate ROS pathway, as a distress, causing not only an increase in antioxidant secondary metabolites and H_2_O_2_ content but also a decrease in the growth of *C. denticulatum*. UV-B with 0.5 W m^−2^ may be the threshold of these two types of UV-B mediated responses. It was also supported by results of metabolites profiles and PLS-DA (Figure 6). However, two types of UV-B mediated responses (UVR8 and ROS pathways) are not mutually exclusive and tend to overlap, depending on UV-B dose thresholds of plant species. Thus, to better understand UV-B mediated responses according to UV-B dose, further studies are required.

In conclusion, our results suggest that 0.25 W m^−2^ UV-B treatment for 6 h a day for 4 days before harvest could be used as a cultivation treatment technique for efficient production of bioactive compounds in *C. denticulatum* grown in PFALs. The application of physical elicitors, such as UV-B light, in PFALs may be available to other medicinal plants used as plant-derived pharmaceuticals.

## 4. Materials and Methods

### 4.1. Plant Materials and Growth Conditions

*C. denticulatum* seeds collected from Pyeongchang, Korea, were sown and grown following the method described by Park et al. [24]. After 3 weeks of sowing, seedlings were transplanted in a PFAL with the following conditions: air temperature 22 ± 0.1 °C, relative humidity 63 ± 0.2%, CO_2_ concentration 627 ± 4.6 µmol mol^−1^, white light-emitting diodes (LEDs) (Figure S1A) photosynthetic photon flux density (PPFD) 200 µmol m^−2^ s^−1^, and light period 16 h. Nutrient solution for *C. denticulatum* (NSC) (electrical conductivity, 2.0 dS m^−1^; pH 5.5) developed in our previous study [24] was supplied to the root zone through capillary wicks inserted in plastic pots filled with the growing medium (Myung-Moon, Dongbu Hannong Co., Seoul, Korea). The nutrient solution was replaced every 2 weeks, and plants were cultivated for 6 weeks.

### 4.2. UV-B Treatment

Five weeks after transplanting, and 3 h after white LEDs were switched on, plants were additionally irradiated with UV-B lamps (Sankyo Ultraviolet Co. Ltd., Kanagawa, Japan), 6 h per day for 6 days (Figure S1B). UV-B energy levels were set by adjusting the number of lamps and the distance between plant canopy and lamps. Different energy levels (0.1, 0.25, 0.5, 1.0, and 1.25 W m^−2^) of UV-B light were calculated as the average of 9 values obtained from equally divided cultivation spaces for each treatment, using a spectroradiometer (JAZ-EL 200, Ocean Optics, Dunedin, FL, USA) at the level of the plant canopy. The different energy levels (W m^−2^), daily radiant energy (kJ d^−1^), and total radiant energy (kJ) for each UV-B treatment are presented in Table 2.

### 4.3. Shoot Biomass

Shoots were harvested on days 2, 4, and 6 of the UV-B treatment, and shoot fresh and dry weights were measured to assess changes in shoot growth according to the applied energy levels of UV-B light. After two hours after UV-B lamps were turned off, the shoot and root were separated and collected at the basal end of plants. Shoot fresh weight was measured using an electronic scale (Si-234, Denver Instrument, Bohemia, NY, USA) and shoot dry weight was measured after freeze-drying at −75 °C for over 72 h using a lyophilizer (Alpha 2-4 LSCplus, CHRIST, Osterode am Harz, Germany).

### 4.4. Maximum Quantum Yield of Photosystem II (Fv/Fm)

One hour after UV-B lamps were turned off, Fv/Fm was measured daily, using a chlorophyll fluorescence meter (PAM 2000, Heinz Walz GmbH, Effeltrich, Germany), to investigate the effects of different energy levels of UV-B treatment on the electron transport system of PSII during the light reaction of photosynthesis. Prior to this measurement, *C. denticulatum* plants were adapted to the dark for 30 min, and then similar-sized leaves of five plants per treatment were used. Maximum fluorescence (Fm) and minimum fluorescence (Fo) were obtained using a 20 kHz saturating light pulse with 1100 µmol m^−2^ s^−1^ PPFD, and Fv/Fm was then calculated using the following equation: Fv/Fm = (Fm − Fo)/Fm.

### 4.5. H_2_O_2_ Content

Leaf tissue samples (0.2 g), collected on days 2, 4, and 6 of the UV-B treatment, were rapidly frozen with liquid nitrogen and stored in a deep-freezer at −70 °C. Leaf tissues were powdered in liquid nitrogen using a pre-chilled pestle and mortar, and the powder was then mixed with 2 mL of 100 mM potassium phosphate buffer (pH 7.4). The homogenate was transferred to 2 mL microtube, and centrifuged at 15,000× *g* for 20 min at 4 °C. The supernatant was filtered through a 0.22 µM syringe filter and the final solution was used to analyze H_2_O_2_ content, using a hydrogen peroxide assay kit (DG-PER500, DoGenBio, Seoul, Korea). The absorbance of final samples was measured at 560 nm, using a multi-mode reader (Synergy HTX, BioTek Instruments, VT, USA).

### 4.6. Total Chlorophyll and Carotenoid Contents

For chlorophyll content analysis, powdered samples (40 mg) were mixed with 3 mL acetone (80%, *v*/*v*), and the mixture was vortexed and sonicated for 25 min to extract chlorophylls. Following centrifugation at 15,000× *g* for 2 min, the supernatant was diluted 4-fold with acetone (80%, *v*/*v*). The absorbance of the final solution was measured using a spectrophotometer (UV-1800, Shimadzu, Kyoto, Japan) at 663.6, 646.6, and 750 nm, and chlorophyll a, chlorophyll b, and chlorophyll a + b were calculated using the equation described by Porra et al. [43].

To analyze total carotenoid content, 100 mg of the powdered samples were mixed with 1 mL aqueous ethanol (70%, *v*/*v*) and sonicated for 90 min. The supernatant obtained after centrifugation at 15,000× *g* for 2 min was diluted 4-fold with aqueous ethanol (70%, *v*/*v*), and total carotenoid content was analyzed and calculated according to the method described by Sumanta et al. [44].

### 4.7. Total Phenolic Content and Antioxidant Capacity

Frozen leaf sample (0.2 g) was powdered in liquid nitrogen using a pre-chilled pestle and mortar and mixed with 3 mL acetone (80%, *v*/*v*) and sonicated for 15 min. The mixture was incubated for 12 h at 4 ℃ and −20 °C for analyzing total phenolic content and antioxidant capacity, respectively. Total phenolic content was analyzed according to the Folin-Ciocalteu method as described in Park et al. and Ainsworth et al. [24,45]. Extract sample (50 μL) was mixed with 135 mL distilled water, 750 mL 10% (*v*/*v*) Folin-Ciocalteau reagent (Sigma-Aldrich, St. Louis, MO, USA), 600 mL 7.5% (*w*/*v*) Na_2_CO_3_ (Samchun, Seoul, Korea). The absorbance of the final mixture was measured at 765 nm using a spectrophotometer (UV-1800, Shimadzu, Kyoto, Japan). Total phenolic content was represented as the content of gallic acid (mg) per unit dry weight.

Antioxidant capacity was determined by ABTS (aminobenzotriazole; 2,2′-azino-bis (3-ethyl benzothiazoline 6-sulfonic acid) diammonium salt) method as described in Park et al. and Miller and Rice-Evans [24,46]. ABTS (Sigma-Aldrich, St. Louis, MO, USA) was mixed with 5 mMphosphate buffered saline (PBS). The absorbance of the mixture was adjusted to 0.7 at 730 nm. Then, extract sample (100 μL) was added to 1 mL of mixture. After 1 min, the absorbance of the final mixture was measured. Antioxidant capacity was represented as trolox (6-Hydroxy-2,5,7,8-tetramethylchromane-2-carboxyl acid) (mM) (Sigma-Aldrich, St. Louis, MO, USA) per unit dry weight.

### 4.8. Hydroxycinnamic Acids (HCAs)

The freeze-dried samples were used to analyze the content of HCAs, including caftaric acid, chlorogenic acid, 3,5-DCQA, and chicoric acid. Powdered samples (100 mg) were mixed with 1 mL aqueous ethanol (70%, *v*/*v*) and the solution was sonicated for 90 min. Four HCAs were analyzed by high-performance liquid chromatography (YL9100, Young Lin Instrument Co., Ltd., Anyang, Korea), as described by Park et al. [24]. Standard samples of caftaric acid (ChemFaces, Hubei, China), chlorogenic acid, 3,5-DCQA (Sigma-Aldrich, St. Louis, MO, USA), and chicoric acid (Avention, Incheon, Korea) were used for obtaining the standard curves, and the content of each compound was expressed as mg per unit dry weight.

### 4.9. Metabolite Extraction

*C. denticulatum* samples (50 mg) were extracted twice with 1 mL of 70% aqueous methanol using a mixer mill (Retsch MM400 mixer mill, Retsch GmbH, Haan, Germany) at a frequency of 30 s for 10 min and sonicated in an ultrasonic water bath (Power Sonic 305, Hwashin Technology Co., Seoul, Korea) for 5 min. After extraction, the extracts were centrifuged at 17,000 rpm for 10 min at 4 °C (Universal 320, Hettich GmbH & Co. KG, Tuttlingen, Germany). The supernatants were filtered through a 0.25 μm polytetrafluoroethylene syringe filter and then completely dried using a speed-vacuum concentrator (Modul 4080C, Biotron, Gyeonggi-do, Korea). The final concentration of each sample was adjusted to 20 mg mL^−1^ for mass spectrometry (MS) analysis.

### 4.10. UHPLC-LTQ-Orbitrap-MS Analysis and Data Processing

LC analyses were carried out in an ultra high performance liquid chromatography (UHPLC) system (Vanquish UHPLC, Thermo Fisher Scientific, Waltham, MA, USA) equipped with a vanquish binary pump, an autosampler, a vacuum degasser, and a thermostatic column compartment. Chromatographic separation was performed on a Kinetex C18 column (100 × 2.1 mm i.d, particle size; 1.7 μm, Phenomenex, Torrance, CA, USA), and the injection volume was 5 μL. The column temperature was set to 40 °C and the flow rate was 0.3 mL min^−1^. The mobile phases were purchased from Sigma Aldrich (St. Lousi, MO, USA). The mobile phase consisted of 0.1% formic acid in water (Solvent A) and 0.1% formic acid in acetonitrile (Solvent B) at a flow of 0.3 mL min^−1^. The ratio of mobile phases was maintained at 5% B from 0 to 1 min, 100% B from 1 to 9 min, and sustained at 100% B for 1 min. Then, it was gradual decrease to 5% B over 3 min. The total run time was 14 min. The UHPLC system was coupled to a linear ion trap (LTQ)-Orbitrap mass spectrometer (LTQ Velos pro, Thermo Fisher Scientific, San Jose, CA, USA) equipped with an electrospray ionization source with a Heated Electrospray Ionization-II probe. The ion trap analysis was performed in full-scan ion modes within a range of 100–1500 *m*/*z*. The probe heater and capillary temperatures were set to 300 °C and 350 °C, respectively. The capillary voltage was set to 3.7 kV in a positive mode (negative mode, 2.5 kV). Leucine encephalin was utilized as reference lock mass (*m*/*z* 554.2615). Tandem MS analyses were performed using scan-type turbo data-dependent scanning under the same conditions used for MS scanning.

MS data processing and multivariate statistical analysis were conducted as described in our previous study [47]. UHPLC-LTQ-Orbitrap-MS data were acquired with Xcalibur software (version 2.1, Thermo Fisher Scientific, Waltham, MA, USA). Raw data were converted to a netCDF (*.cdf) format using Xcalibur software. After conversion, the MS data were processed using the Metalign software package (http://www.metalign.nl) to obtain a data matrix containing retention times, accurate masses, and normalized peak intensities. The resulting data were exported to Excel (Microsoft, Redmond, WA, USA) for multivariate data analysis.

### 4.11. Statistical Analysis

Twelve plants per treatment were used for analyzing growth characteristics, total chlorophyll content, total carotenoid content, and individual HCAs content. Six plants per treatment were analyzed for total phenolic content, antioxidant capacity, and H_2_O_2_ content and chlorophyll fluorescence was measured in 5 plants. Analysis of variance was performed in SAS program (Statistical Analysis System, 9.2 Version, SAS Institute, Cary, NC, USA), and significant differences among treatments were determined using Duncan’s multiple range test. Pearson’s correlation coefficients were used to analyze association between the parameters.

Multivariate data analyses were performed using SIMCA-P+ software (version 12.0, Umetrics, Umea, Sweden). PCA and PLS-DA were performed to compare different energy levels of UV-B radiation groups for 4 days. The significance of the PLS-DA model was defined by analysis of variance testing of cross-validated predictive residuals (CV-ANOVA) in the SIMCA-P+ program. The significantly discriminant metabolite with VIP value exceeding 0.7 using the PLS-DA model was represented by box-whisker plots using Statistica, version 7.0 (StatSoft Inc., Tulsa, OK, USA). The metabolites were identified by comparing their retention time, molecular weight, accurate mass, elemental composition, and MS^n^ fragment patterns based on standard compounds and published references.

## Figures and Tables

**Table 1:** Hydroxycinnamic acids (HCAs) contents in *C. denticulatum* subjected to different energy levels of UV-B radiation at 2 and 4 days of treatment. Plants were subjected to UV-B treatments at 5 weeks of transplanting.

Day of Treatment	UV-B Treatment (W m^−2^)	Caftaric Acid	Chicoric Acid	3,5-DCQA	Chlorogenic Acid	Caffeic Acid	Total HCAs
		mg shoot^−1^					
2	0	94	27.3	24.8	10.9	0.16 c ^1^	157 b
	0.1	100	29.8	23.0	12.7	0.26 bc	166 b
	0.25	94	27.1	23.1	11.0	0.29 ab	156 b
	0.5	89	26.3	23.5	11.8	0.21 bc	151 b
	1.0	123	36.4	28.8	15.0	0.39 a	216 a
	1.25	112	29.9	24.9	13.4	0.30 ab	181 ab
4	0	129 b	31.3 b	23.8	13.4	0.18 c	198 b
	0.1	128 b	31.8 b	22.2	11.8	0.22 c	194 b
	0.25	213 a	49.8 a	33.3	17.5	0.52 a	314 a
	0.5	138 b	35.9 b	27.4	15.1	0.33 abc	216 b
	1.0	147 b	35.7 b	23.9	14.0	0.44 ab	221 b
	1.25	168 ab	41.0 ab	28.2	14.1	0.32 bc	251 ab
Significance ^2^	Day	***	***	NS	NS	*	***
	Energy	*	NS	NS	NS	***	*
	D*E	*	*	NS	NS	NS	*

^1^ Different letters indicate a significant difference within each week by Duncan’s multiple range test at * *p* < 0.05. ^2^ The asterisk indicates significant difference by two-way analysis of variance (ANOVA) at * *p* < 0.05 and *** *p* < 0.001 (*n* = 12). NS, not significant.

**Table 2:** Radiation levels and daily radiant energies of UV-B lamps for different energy treatments.

UV-B Treatment	Measured Value	Daily Radiant Energy (kJ d^−1^)	Total Radiant Energy (kJ)
(W m^−2^)			
0.1	0.11 ^1^	2.4	149.7 ^2^
0.25	0.24	5.2	326.6
0.5	0.5	10.8	680.4
1.0	1.01	21.8	1374.4
1.25	1.26	27.2	1714.6

^1^ Average irradiance of UV-B (*n* = 9). ^2^ Integrated radiation energy of UV-B for 6 days.

**Table 3:** Pearson correlation coefficients between shoot fresh and dry weights, Fv/Fm (maximum quantum yield of photosystem II), total chlorophyll content, total carotenoid content, and total HCAs (hydroxycinnamic acids) per unit dry weight and H_2_O_2_ content, total phenolic content, and antioxidant capacity per unit fresh weight of *C. denticulatum* at 4 days of UV-B treatment (0, 0.1, 0.25, 0.5, 1.0, and 1.25 W m^−2^). The asterisk indicates significant difference at * *p* < 0.05, ** *p* < 0.01, and *** *p* < 0.001.

Measured Parameters	Shoot Fresh Weight	Shoot Dry Weight	Total Chlorophyll Content	Total Carotenoid Content	Fv/Fm	H_2_O_2_ Content	Total Phenolic Content	Antioxidant Capacity	Total HCAs
Shoot fresh weight	1.0								
							
Shoot dry weight	0.96 ***	1.0							
	*p* < 0.0001								
Total chlorophyll content	0.17	0.04	1.0						
	*p* < 0.1626	*p* < 0.7625							
Total carotenoid content	−0.16	−0.10	−0.16	1.0					
	*p* < 0.1794	*p* < 0.3910	*p* < 0.1761						
Fv/Fm	0.20	0.02	0.39 *	−0.52 **	1.0				
	*p* < 0.2853	*p* < 0.9027	*p* <0.0345	*p* < 0.0032					
H_2_O_2_ content	−0.05	0.13	−0.28	0.24	−0.56 **	1.0			
	*p* < 0.7823	*p* < 0.4648	*p* < 0.0922	*p* < 0.1632	*p* < 0.0013				
Total phenolic content	−0.16	0.02	−0.51 **	0.34 *	−0.47 **	0.58 ***	1.0		
	*p* < 0.3438	*p* < 0.0947	*p* < 0.0016	*p* < 0.0445	*p* < 0.01	*p* < 0.0003			
Antioxidant capacity	−0.17	−0.001	−0.42 *	0.40 *	−0.41 *	0.44 **	0.90 ***	1.0	
	*p* < 0.3320	*p* < 0.9925	*p* < 0.0113	*p* < 0.0152	*p* < 0.0256	*p* < 0.0075	*p* < 0.0001		
Total HCAs	−0.17	−0.13	−0.22	0.47 ***	−0.27	0.06	0.47 **	0.57 ***	1.0
	*p* < 0.1469	*p* < 0.2726	*p* < 0.0596	*p* < 0.0001	*p* < 0.1479	*p* < 0.7427	*p* < 0.004	*p* < 0.0003	

## Supplementary Materials

Supplementary materials can be found at https://www.mdpi.com/1422-0067/21/19/7134/s1. Figure S1. Relative spectral distributions white LEDs (A) and a UV-B lamps (B). Table S1. Chemical characteristics of C. denticulatum subjected to different energy levels of UV-B radiation at 4 days after treatment using UPLC-LTQ-Orbitrap-MS.

## Abbreviations

HCAs	Total hydroxycinnamic acids
PLS-DA	Partial least squares discrimination analysis
Fv/Fm	Maximum quantum yield of PSII
ROS	Reactive oxygen species
UVR8	UV resistance locus8
*C. denticulatum*	*Crepidiastrum denticulatum*
PFALs	Plant factories with artificial lighting
PSII	Photosystem II
3,5-DCQA	3,5-di-O-caffeoylquinic acid
UHPLC-LTQ-Orbitrap-MS	Ultra high performance liquid chromatography-linear ion trap-Orbitrap mass spectrometer
PCA	Principal component analysis
MS	Mass spectrometry
QC	Quality control
NPQ	Non-photochemical quenching
Q_A_	Quinone
COP1	CONSTITUTIVELY PHOTOMORPHOGENIC1
HY5	ELONGATED HYPOCOTYL5
HCTs	Hydroxycinnamoyl transferases
PPFD	Photosynthetic photon flux density
NSC	Nutrient solution for *Crepidiastrum denticulatum*
Fm	Maximum fluorescence
Fo	Minimum fluorescence
ANOVA	Analysis of variance
TPS	Terpene synthase activity
