# Supplementary material for: Physiologic and Metabolic Changes in Crepidiastrum denticulatum According to Different Energy Levels of UV-B Radiation
Source: Int J Mol Sci. 2020 Sep 27;21(19):7134. doi: 10.3390/ijms21197134 (PMC7582291; doi:10.3390/ijms21197134)
Supplement: Supplementary file 1 [file ijms-21-07134-s001.pdf]

Table S1. Chemical characteristics of *C. denticulatum* subjected to different energy levels of UV-B radiation at 4 days after treatment using UPLC-LTQ-Orbitrap-MS.

NO.	RT (min)	Tentative metabolites[a]	Measured *m/z*		MS^n fragment	M.W.	M.F.	Error (ppm)	*p*-value	Ref.
			negative	positive						
1	0.84	Quinic acid	191.0565	-	191>172,111	192	$C_7H_{12}O_6$	2.1	0.087	[1],
2	2.18	Caftaric acid	311.0409	335.0375	311>179, 149>131	312	$C_{13}H_{12}O_9$	0.9	0.088	[1]
3	4.96	Luteolin-7-O-b-D-glucoside	447.0945	449.1067	447>285,241>198	448	$C_{21}H_{20}O_{11}$	2.2	0.004	[2]
4	4.97	Luteolin-7-O-b-D-glucuronide	461.0735	463.0855	461>285,241>198	462	$C_{21}H_{18}O_{12}$	1.2	0.000	[1]
5	5.09	11β,13-Dihydroixerin Z	423.1696	425.1798	423>243	424	$C_{21}H_{28}O_9$	1.5	0.009	[3]
6	5.15	Di-O-caffeoylquinic acid	515.1196	517.3142	515>353>191	516	$C_{25}H_{24}O_{12}$	0.1	0.243	[1],[2]
7	5.78	N. I.	423.2240	447.2202	423>291>161	424	-	-	0.000	
8	5.91	Youngiaside B	557.2045	581.1990	557>423,243>199	558	$C_{29}H_{34}O_{11}$	1.2	0.000	[2]
9	6.39	N. I.	379.1982	403.1833	379>335>291	380	-	-	0.000	
10	6.48	Ixerin U	-	587.2149		588	$C_{30}H_{36}O_{12}$	2.5	0.326	[4]
11	6.55	N. I.	413.2196	437.2156	-	414	-	-	0.000	
12	6.71	Ixerochinoside	691.2408	715.2359	691>447>175	692	$C_{37}H_{40}O_{13}$	1.7	0.000	[3], [5]
13	7.90	N. I.	675.3613	699.3546	675>397>235	676	-	-	0.000	
14	8.58	N. I.	559.3134	-	-	560	-	-	0.000	
15	8.95	N. I.	325.1847	-	-	324	-	-	0.001	
16	9.40	N. I.	339.2007	-	-	340	-	-	0.000	

RT, retention time; M.W., molecular weight; M.F., molecular formula; Ref., Reference; [a] Identified metabolites based on VIP > 0.7 both PLS 1 and PLS 2 by PLS-DA; Metabolites were tentatively identified by matching molecular weight and formula, MS^n, and references.

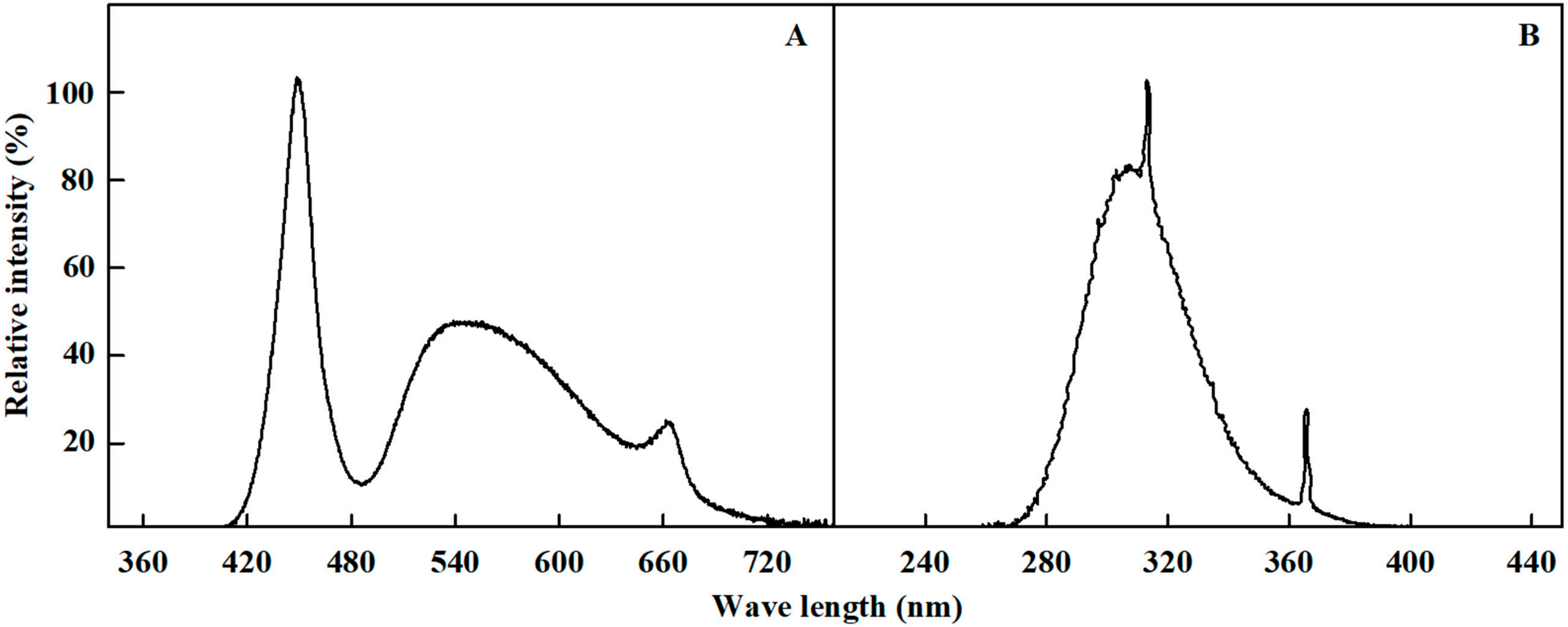

Figure S1. Relative spectral distributions white LEDs (**A**) and a UV-B lamps (**B**).

References

1. Lee, H.J.; Cha, K.H.; Kim, C.Y.; Nho, C.W.; Pan C.H. Bioavailability of hydroxycinnamic acids from *Crepidiastrum denticulatum* using simulated digestion and Caco-2 intestinal cells. *J. Agric. Food Chem.* **2014**, 62, 5290-5292.

2. Kim, M.S.; Park, Y.G.; Lee, H.J.; Lim, S.J.; Nho, C.W. Youngiasides A and C isolated from youngia denticulatum inhibit UVB-induced MMP expression and promote type I procollagen production via repression of MAPK/AP-1/NF-κB and activation of AMPK/Nrf2 in HaCaT cells and human dermal fibroblasts. *J. Agric. Food Chem.* **2015**, 63, 5428-5438.

3. Na, Z.; Cho, J.Y.; Lee, H.J.; Chung, J.H.; Park, K.D.; Lee, Y.J.; Shin, S.C.; Rim, Y.S.; Park, K.H.; Moon, J.H. Spectral assignments and reference data. *Magn. Reson. Chem*. **2007,** 45, 275-278.

4. Seto, M.; Miyase, T.; Fukushima, S. Sesquiterpene lactones from *Ixeris dentate* NAKAI. *Chem. Pharm. Bull.* **1986**, 34, 4170-4176.

5. Khalil, A.T.; Shen, Y.C.; Guh, J.H.; Cheng, S.Y. Two new sesquiterpene lactones from *Ixeris chinensis*. *Chem. Pharm. Bull.* **2005**, 53, 15-17.

6. Park, S.A.; Son, S.Y.; Lee, A.Y.; Park, H.G.; Lee, W.L.; Lee, C.H. Metabolite profiling revealed that a gardening activity program improves cognitive ability correlated with BDNF levels and serotonin metabolism in the elderly. *Int. J. Environ. Res. Public Health* **2020**, 17, 541.
